# Chinese expert consensus on prevention, diagnosis, and management of venous thromboembolism in adult burn patients (2024)

**DOI:** 10.1186/s40779-025-00653-9

**Published:** 2025-11-02

**Authors:** Feng Zhu, Xiao-Bin Liu, Wei Zhou, Tuo Shen, Qi-Min Ma, Yu-Song Wang, Wen-Bin Tang, Xiao-Jian Li, Xi Yin, Ding-Hong Min, Hao Li, Shi-Hui Zhu, Yue-Sheng Huang, Guang-Hua Guo, Xiao-Bing Fu, Burn and Trauma Branch of the Chinese Geriatric Medical Association, and Critical Care Group of Burn Surgery Branch of the Chinese Medical Association

**Affiliations:** 1 Department of Critical Care Medicine, Shanghai East Hospital, School of Medicine, Tongji University, Shanghai, 200120 China; 2 Department of Burn Surgery, the First Affiliated Hospital of Naval Medical University, Shanghai, 200433 China; 3 Department of Burns and Plastic Surgery, Guangzhou Red Cross Hospital of Jinan University, Guangzhou, 510000 China; 4 Department of Burns, Zhangjiagang First People’s Hospital, Suzhou, 215638 Jiangsu China; 5 Medical Center of Burn Plastic and Wound Repair, the First Affiliated Hospital of Nanchang University, Nanchang, 330006 China; 6 Department of Burns and Plastic Surgery, Shanghai Children’s Medical Center, Shanghai Jiaotong University, Shanghai, 200127 China; 7 Department of Wound Repair, Institute of Wound Repair and Regeneration Medicine, Southern University of Science and Technology Hospital, Southern University of Science and Technology School of Medicine, Shenzhen, 518055 Guangdong China; 8 Key Laboratory of Wound Repair and Regeneration of PLA, the Fourth Medical Center of PLA General Hospital, Beijing, 100048 China

**Keywords:** Burn, Adult, Venous thromboembolism (VTE), Diagnosis, Management, Prevention, Consensus, Deep vein thrombosis (DVT), Pulmonary thromboembolism (PE), Anticoagulation

## Abstract

Venous thromboembolism (VTE) management in adult burn patients has become a crucial focus in China. The intricate nature of VTE necessitates specialized anticoagulation strategies due to the unique challenges posed by burn injuries. To address this pressing issue, the Burn and Trauma Branch of the Chinese Geriatric Medical Association and Critical Care Group of Burn Surgery Branch of the Chinese Medical Association organized a panel of domestic experts in burn surgery, critical care medicine, vascular surgery, nursing, and health statistics and methodology from Chinese hospitals to discuss VTE-related issues in burn injury, the heightened risk factors such as extensive tissue damage and prolonged immobilization, and the delicate balance required in anticoagulation therapy to mitigate bleeding risks. Based on the latest available research evidence as well as the clinical experience of the panel experts, this consensus comprehensively evaluates factors such as generalizability, suitability, and the potential implications for resource allocation. It also appropriately weighs the clinical advantages against possible drawbacks, resulting in the formulation of 21 guideline recommendations.

*Registration* Practice Guideline REgistry for transPAREncy (PREPARE): No. 2023CN656.

**Supplementary Information:**

The online version contains supplementary material available at 10.1186/s40779-025-00653-9.

## Background

Venous thromboembolism (VTE), which consists of pulmonary embolism (PE) and deep-vein thrombosis (DVT), is becoming a common issue in most countries [1]. The incidence of VTE in Europe and the USA is estimated to be 1–2 per 1000 person-years, but varies widely by age, sex, race, and medical conditions [2]. In Asia, the rates of VTE are thought to be lower than in Europe and the USA [3]. In China, data from 90 hospitals between 2007 and 2016 showed that the hospitalization rate of VTE has increased from 3.2 per 100,000 to 17.5 per 100,000 over the past 10 years [4]. According to the DissolVE-2 study, despite the incidence of surgical VTE was 9.3%, and that of medical VTE was 6.0% in China after reasonable precautions were taken, the proportion of medium and high risk of VTE in surgical hospitalized patients was 32.7% and 53.4% respectively, and the proportion of high risk of VTE in medical patients was 36.6% [5].

VTE is a major cause of morbidity and mortality. The complications of VTE may be significant and morbid. Acute PE may be fatal. Chronic thromboembolic pulmonary hypertension may complicate acute or recurrent PE. Post-thrombotic syndrome occurs commonly following DVT because of the development of deep venous reflux and/or residual venous obstruction. Post-thrombotic syndrome is associated with skin changes and ulceration and causes an adverse impact on quality of life and escalation of health care costs [6]. The increasing burden of VTE in China over the past decade and the serious lack of prevention have also become increasingly prominent. The lifetime risk of VTE keeps increasing with age among adults [7]. VTE is common and associated with significant morbidity and mortality [8].

The risk of VTE escalates remarkably in burn patients due to factors, including tissue damage from shock, resuscitation, hypothermia, intense stress responses, inflammation, vascular endothelial injury, and the diminution or dilution of coagulation factors [9]. Additional risks such as prolonged immobility, repetitive vascular punctures, fever, malnutrition, and the stresses inherent in long-term treatments like surgery and frequent dressing changes post-burn [10–12]. However, because of the lack of high-quality clinical research and limited basic research, there are still great differences in the occurrence and development, clinical evaluation, prevention, and management of VTE, and some even have gaps in worldwide of burns.

China is a populous country with the highest incidence of burns in 2019 (approximately 1,079,670 cases), accounting for 12% of global total [13]. Domestic relevant research reports show that the incidence of VTE in burn patients is 1.04% to 3.9% [14, 15]. A wealth of clinical experience has been accumulated through previous medical practices. In recent years, as clinical understanding of VTE in burn patients has deepened and related disciplines have made progress in this field, Chinese burn practitioners increasingly recognize the importance of VTE in burn prevention and treatment, which significantly impacts the prognosis and quality of life for such patients. Currently, there are no consensus or guideline documents on the prevention, diagnosis, and management of VTE in burn patients both domestically and internationally. To address the gap in this field, the Burn and Trauma Branch of the Chinese Geriatric Medical Association and Critical Care Group of Burn Surgery Branch of the Chinese Medical Association have collaboratively provided contemporary information on VTE in adult burn patients and developed this consensus. In this consensus, we focus on adult burn patients as this is the primary population for burn care with more evidence and experience available. Evidence and experience regarding VTE in pediatric and special population burns (the elderly, especially more than 90 years old, pregnant women and so on) are relatively limited and more complex, which will not be discussed here. This consensus is not only grounded in the currently limited evidence-based literature, but also incorporates valuable insights from clinical studies, guidelines, and consensus documents across relevant disciplines over the past decade. We carefully reviewed domestic and international data on the prevention, diagnosis, and management of VTE in adult burn patients, based on the latest available research evidence as well as the clinical experience of the panel experts. This consensus comprehensively evaluates factors such as generalizability, suitability, and the potential implications for resource allocation. It also appropriately weighs the clinical advantages against possible drawbacks, resulting in the formulation of 21 guideline recommendations, aiming to provide scientific and comprehensive guidance for clinical practice.

## Methods

### Questionnaire design and main content

A questionnaire design team was established. The questionnaire focused on the high-risk factors, prevention, diagnosis, and treatment of VTE after burns, and deconstructed clinical problems according to the PICO principle (P: population/patients, I: intervention measures, C: control/comparison, O: outcome indicators) [16]. The literature search team systematically retrieved and organized studies on VTE prevention, diagnosis, and management in adult burn patients from databases including PubMed, WOS, and CNKI, while the evidence evaluation team rigorously assessed and classified the evidence to ensure scientific validity. The drafting team subsequently developed clinical questions, formulated preliminary opinions, and designed the first-round Delphi questionnaires.

The questionnaire survey comprised 3 main aspects with 21 items, covering high-risk factors for VTE after burn, assessment and diagnosis of VTE after burn, and comprehensive management of VTE after burn, including medication, physical therapy, and surgery. The questionnaire included the basic information of the experts, their recognition of all listed items, their familiarity degree with various aspects, the basis of their judgment, and the degree of influence. Meanwhile, to facilitate expert feedback for potential revisions, the questionnaire included supplementary materials for expert suggestions.

### Expert selection

This consensus was under the guidance of the Burn and Trauma Branch of the Chinese Geriatric Medical Association, and Critical Care Group of Burn Surgery Branch of the Chinese Medical Association. A total of 37 experts panel, comprising esteemed professionals in burn surgery, critical care medicine, vascular surgery, nursing, and health statistics and methodology from Chinese hospitals and scientific research institutions, were screened and rigorously evaluated each consensus item. All participants were required to complete a conflict-of-interest declaration to ensure transparency and integrity in the consensus process.

### The process of consensus formulation

**Fig. 1:**
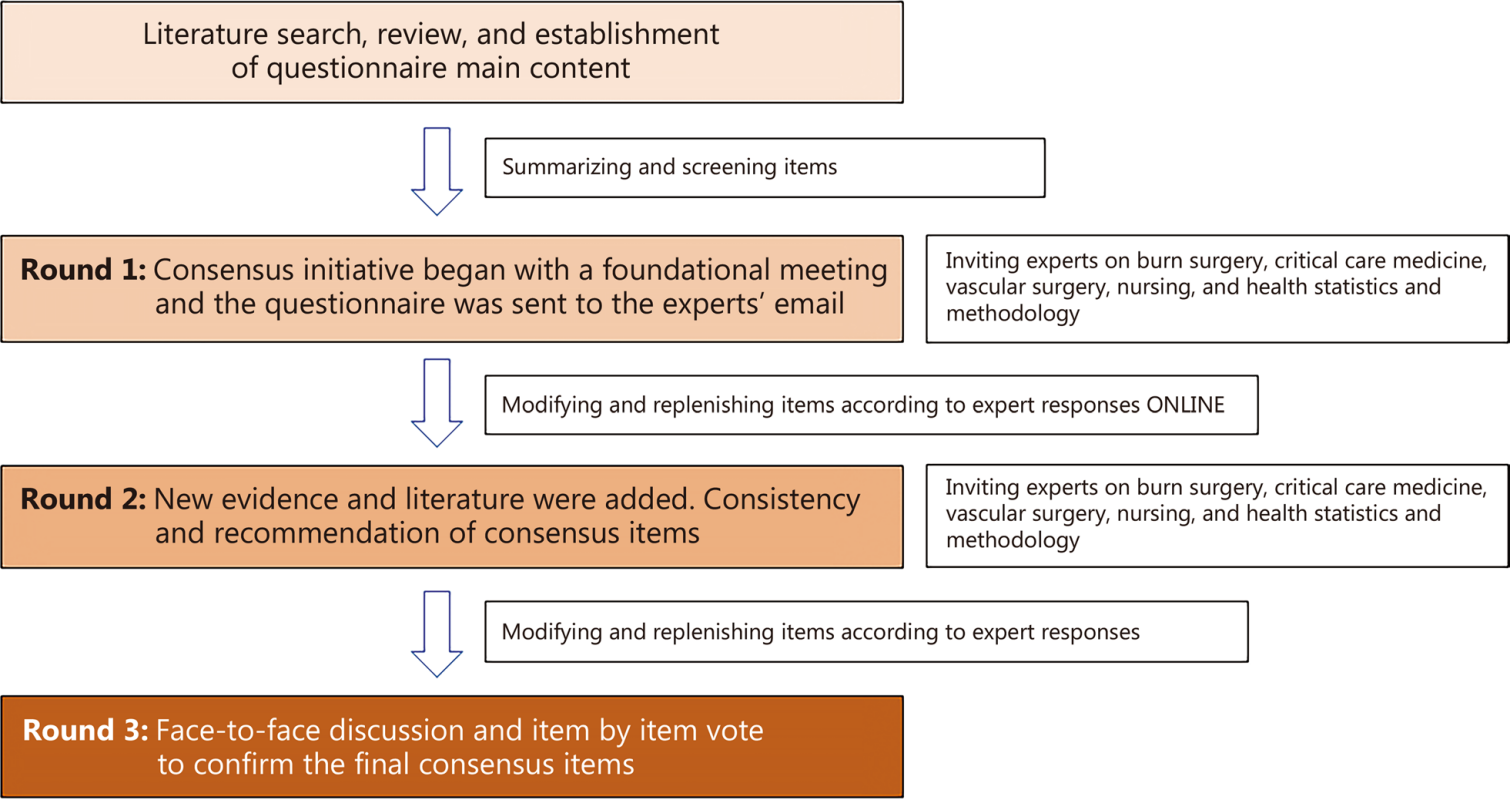
Flowchart Delphi process

Initiated in September 2021, the consensus initiative began with a foundational meeting and the questionnaire was sent to the relevant experts’ email addresses. In June 2022, due to the COVID-19 epidemic, the first draft of the consensus, according to the questionnaire feedback, was reported online. According to the survey feedback, the writing team made statistical analysis of each recommendation and modified and improved the expression and citation of evidence. In July 2023, the second consensus report was made in-person meetings and new evidence and literature have been added. During the second round of discussions, content with disputes or new proposals underwent a second round of discussions and Delphi surveys. Subsequently, the draft was registered on the International Practice Guidelines Registry Platform (2023CN656). In March 2024, the third report of the consensus was discussed item by item and voted on by all members, and finalized (Fig. 1).

### Literature search and evaluation

Comprehensive searches were conducted in databases such as PubMed, Embase, Cochrane Library, Epistemonikos, China Biology Medicine, Wanfang, and CNKI, focusing on systematic reviews, meta-analyses, and network meta-analyses. Sixty-seven original studies were also reviewed, including randomized controlled trials (RCTs), cohort studies, and epidemiological investigations, up to January 1, 2023. The grading of evidence and recommendations was aligned with the Grading of Recommendations Assessment, Development and Evaluation (GRADE) criteria [17, 18].

Experts complete the Delphi questionnaires based on evidence grade and clinical experience. There were 5 options: “recommended”, “somewhat recommended”, “neutral”, “somewhat not recommended”, and “not recommended”, and supplementary text opinions could be added. Based on feedback from rounds of surveys, the draft team conducted statistical analysis to calculate the agreement rate (combined percentage of “strongly recommended” and “somewhat recommended” responses) for each recommendation. They then revised and refined both the wording and evidence references. Recommendations with an agreement rate ≥ 90% were marked as strongly recommended, those between 70% and 89% as moderately recommended, and those below < 70% not included.

The risk of bias and quality assessment followed internationally recognized evaluation standards, utilizing the Revised Cochrane risk of bias tool version 2 (RoB 2) for randomized studies and the Risk of Bias in Non-randomized Studies—of Interventions (ROBINS-I) for non-randomized studies [19, 20]. The guideline development followed the WHO’s 2014 Guideline Development Manual [21].

### Statistical analysis

Statistical analysis was performed with R 4.4.1. The following indicators were calculated: (1) the positive coefficient of experts, defined as the questionnaire recovery rate; (2) the expert judgment coefficient (Ca) for each item, the familiarity coefficient (Cs), and the authority coefficient (Cr) [Cr = (Ca + Cs)/2], with all results presented as mean ± standard deviation (SD); (3) the mean score ± SD, full score ratio (%), and coefficient of variation (CV, defined as SD divided by the mean score) for each item. The Kendall’s coordination coefficient was also calculated for each recommendation category to evaluate the consistency of expert scoring. To ensure the reliability, authority, and consistency of expert ratings, the following threshold criteria were established: questionnaire response rate ≥ 70% indicates good expert participation [22]; Cr ≥ 0.70 signifies high expert authority and credible judgment [23]; CV ≤ 0.25 indicates that expert opinions are concentrated and exhibit good consistency [24]; full‐score ratio ≥ 50% suggests that a significant proportion of experts highly recognize the item [25]; Kendall’s W close to 1 indicates strong consistency, and a W in the range 0.30 to 0.60 is considered medium to strong consistency (*P* < 0.05) [26]. If multiple of these standards are not met, the research team will consider revising or discarding the item in subsequent Delphi rounds. The Research ANd Development (RAND)/University of California, Los Angeles (UCLA) Appropriateness Method (RAM) was applied to evaluate both the consistency of expert scoring and the strength of the recommendations.

## Results

### Statistical results

#### Basic information of experts

A total of 37 experts in the field of burn surgery, critical care medicine, vascular surgery, nursing, and health statistics and methodology were selected, and all 37 questionnaires were returned. Among the participating experts, 16.2% are under 45 years old, 54.1% are between 45 and 60 years old, 29.7% are over 60 years old, 27.0% have less than 20 years of working experience, 32.4% have between 20 and 30 years of working experience, and 40.6% have over 30 years of working experience (Additional file 1: Table S1). The basic information of consultant experts is provided in Additional file 1: Consultant experts.

#### Degree of enthusiasm in experts

All 37 questionnaires were recovered with a recovery rate of 100.0%, indicating that the selected experts were highly motivated and actively engaged in the consensus process. Across all 21 recommendations, the CVs ranged from 0.03 to 0.09, reflecting a very high level of agreement among experts. The Kendall’s correlation coefficients ranged from 0.27 to 0.32, all statistically significant (*P* < 0.05), demonstrating satisfactory concordance in expert scoring, indicating a statistically significant but moderate level of agreement among experts in this round of the survey.

#### Expert authority

The Cr for each recommendation was calculated as the average of the Ca and the Cs, both expressed as mean ± SD. In this study, Cr values ranged from 0.81 ± 0.06 to 0.86 ± 0.04 across all items, reflecting a high degree of expert authority. Similarly, the Ca ranged from 0.81 ± 0.06 to 0.85 ± 0.04, and the Cs ranged from 0.82 ± 0.06 to 0.87 ± 0.03. These results support the reliability and credibility of the consensus process.

#### Consensus evaluation and recommendation strength

Based on the CV for each recommendation, 3 recommendations were rated as “perfect consistency” (CV ≤ 0.04), 10 recommendations as “very good consistency” (0.05 ≤ CV ≤ 0.07), and 8 recommendations as “good consistency” (0.08 ≤ CV ≤ 0.10). Regarding recommendation strength, 18 items were classified as strong recommendations, and 3 items as weak recommendations, with no items rated as “no recommendation”. These findings directly correspond to the 21 finalized consensus recommendations for VTE prevention, diagnosis, and management in adult burn patients. The concentration, coordination, and authority of the 21 consensuses are detailed in Additional file 1: Table S2.

### Recommendation 1: VTE is a prevalent complication following burn injuries, particularly in patients with extensive, critical burns. This condition poses significant risks and warrants early attention and both mechanical and pharmacologic preventive interventions (Strong recommendation, evidence level: high)

Despite the absence of precise epidemiological data delineating the full scope of VTE prevalence among burn patients, existing literature provides evidence for an elevated risk in this cohort, especially in those with extensive critical burns. This risk was first systematically documented by Foley et al. [27] in 1968. In their seminal autopsy study of 233 deceased burn patients, microthrombosis was observed in a quarter of the specimens, with 0.8% of deaths attributable to PE. Subsequent retrospective studies, primarily focused on symptomatic patients, reported variable VTE incidence ranging from 0.25% to 5.92% [28–33]. A comprehensive retrospective study by Fecher et al. [28] encompassing 4102 burn patients over a decade, wherein all individuals received prophylactic-dose subcutaneous heparin and underwent imaging for symptomatic evaluation, revealed a DVT occurrence rate of 0.25%. In a pivotal study by Mullins et al. [32], employing Doppler ultrasound (DUS) for patients subjected to prolonged mechanical ventilation and immobilization, DVT was diagnosed in 86 out of 1452 burn patients, resulting in an incidence of 5.92%, predominantly in the lower extremities. Moreover, an analysis by Satahoo et al. [34] in 2015, involving 36,638 burn patients from the United States National Burn Database, identified a DVT incidence of 0.8%. Recently, Lu et al. [35] conducted a study in Australia and New Zealand, encompassing data from 1475 burn patients registered between 2013 and 2018, and reported a VTE incidence of 1.36%. Consistently, retrospective studies in China have reported DVT incidences of 1.04% [14] and 0.98% [36], respectively. Overall, these data indicate a relatively low incidence of VTE among burn patients. However, due to inherent differences in study designs, inclusion criteria, and diagnostic approaches, the reported incidence rates of VTE in burn patients vary substantially across retrospective and prospective studies [33]. Retrospective analyses often yield lower incidence rates compared to their prospective counterparts. The reported incidence of VTE in prospective studies ranges considerably, from 6.08% to 23.3% [37–39].

The American Burn Association (ABA) conducted a large study including 33,637 thermally injured patients between 1995 and 2007 [21]. The overall incidence of VTE post-burn was 0.61%, comprising DVT at 0.48% and PE at 0.18%. Notably, among patients with TBSA > 10%, the incidence increased to 1.22%, highlighting the higher VTE risk in patients with more extensive burns [40]. An illuminating prospective study by Wahl et al. [38] reported a notably high incidence of DVT at 23.3% among adult burn patients in their burn center.

Up to 50% of DVT cases in burn patients may be asymptomatic [41], and burn-related symptoms such as swelling and pain can obscure DVT signs, complicating diagnosis, particularly in severe cases requiring mechanical ventilation. Therefore, the true incidence of DVT in burn patients is likely higher than reported, as many cases are asymptomatic and difficult to detect. Untreated DVT can lead to PE, which may be fatal; however, estimates of mortality vary widely depending on patient population and study methodology [42, 43]. This underscores the importance of early diagnosis and proactive DVT prevention.

Notably, postmortem examinations reveal even more striking data, with incidences of DVT climbing as high as 60% in certain studies, significantly surpassing those observed in retrospective analyses [27, 44, 45].

Global research consistently demonstrates that early prevention of VTE plays a crucial role in substantially lowering its incidence among burn patients. Following the introduction of preventive interventions, the proportion of patients developing VTE post-burn decreased to between 50% and 84% in countries including Canada, United States, and United Kingdom [46–48]. A Chinese study demonstrated that a standardized VTE prevention program markedly lowered VTE incidence compared to traditional methods (1.56% vs. 10.17%) [15]. These findings emphasize the importance of early VTE prevention in improving care and outcomes for burn patients.

### Recommendation 2: Advanced age, obesity, extensive burns (especially on lower extremities), deep venous catheters, prolonged intensive care unit (ICU) stay, long-term immobility in bed, wound infection, number of operations, and the presence of inhalation injuries or trauma should be recognized as primary risk factors for VTE following burns (Strong recommendation, evidence level: medium)

In non-burn critically ill patients, numerous studies have pinpointed factors like advanced age and obesity as VTE risk factors [49–52]. However, research specifically focused on VTE in burn patients remains comparatively scarce. The American College of Chest Physicians-8th edition (ACCP-8) antithrombotic guidelines [53] recommend routine VTE preventive interventions in burn patients with additional risk factors such as advanced age, morbid obesity, extensive or lower extremity burns, concomitant lower extremity trauma, femoral vein catheter usage, and/or prolonged immobilization.

Progressing clinical research has identified several risk factors for VTE in burn patients, including surgery or frequent dressing changes, mechanical ventilation, central venous catheters, wound infection, and associated trauma in addition to those risk factors above [32, 33, 36, 54, 55]. A retrospective study from the National Burn Database in the United States, involving 33,637 burn patients, revealed that factors like advanced age, having more than 2 comorbidities, concurrent inhalation injuries, extended ICU stays and ventilation (over 48 h), increased ventilation days, more surgical interventions, and an extensive burn area, significantly escalate VTE risk. Further analysis identified burn total body surface area (TBSA), ICU stay duration, and the number of surgeries as independent risk factors for heightened VTE risk [56]. A retrospective analysis of 845 burn patients in China indicated that advanced age, lower extremity burns, wound infection, femoral vein catheterization, and prolonged bed rest (over 40 d) were independent VTE risk factors. Of these, advanced age, wound infection, femoral vein catheterization, and extended bed rest emerged as strong independent predictors [54]. In the analysis from Pannucci et al. [40], 3 factors including TBSA burned, ICU days, and number of operations, were independently associated with increased VTE risk after controlling for age, gender, presence of inhalation injury, central venous catheter insertion, and ventilator days.

### Recommendation 3: Caprini risk score (CRS) is recommended for VTE risk assessment in hospitalized burn patients (Strong recommendation, evidence level: medium)

VTE risk assessment is essential for all hospitalized patients, and specific validated scoring systems are recommended according to patient type. For example, the CRS is widely used in surgical and burn patients, the Autar score is applied in general medical inpatients, and the Well’s grading scale is primarily used for patients with suspected DVT or PE. This stratified approach ensures that risk assessment is tailored to the clinical context, facilitating appropriate preventive interventions.

The CRS was originally developed by Caprini et al. [57] at Northwestern University in 1991 and underwent multiple revisions, reaching its mature form in 2010 [58–60]. The ACCP-9 evaluated the CRS, noting its simplicity and reasonable risk stratification, although it was not derived using rigorous statistical methods [61]. Nevertheless, the CRS remains a thoroughly validated tool for predicting surgery-related and burn-related VTE risk, and its use is strongly recommended in these patient populations.

CRS comprehensive coverage of surgical scenarios makes it particularly suitable for surgical patients. It encompasses 40 different thrombosis risk factors, covering virtually all potential VTE risks in hospitalized medical and surgical patients (including cardiac, respiratory, psychiatric, orthopedic, general surgery, obstetrics, and gynecology departments). Factors are scored from 1 to 5 based on severity, with the total score determining the patient’s risk category: low risk (0–1 point), medium risk (2 points), high risk (3–4 points), and extremely high risk (≥ 5 points) [57]. This scoring system offers a comprehensive approach to assessing the VTE risk in a diverse patient population. As previously discussed, factors like hypercoagulation, vascular integrity destruction, and central venous catheterization in burn patients are all independent predictors of VTE, making the CRS theoretically suitable for evaluating VTE risk in burn surgery patients.

A single-center study involving 1939 burn patients assessed using the CRS found that the incidence of symptomatic VTE was 0.18% for CRS of 0–2 points, 0.69% for CRS of 3–4 points, 0.78% for CRS of 5–6 points, 3.66% for CRS of 7–8 points, and 8.82% for CRS > 8 points [62]. Notably, when the CRS > 8 points, the incidence of DVT soared to 62% [63]. This study supports the use of the CRS for informed decision-making regarding VTE prevention strategies in burn patients. A retrospective study by Peng et al. [54] assessed DVT risk using the CRS. The patients were categorized into non-DVT (*n* = 830) and DVT (*n* = 15) groups. Among 360 (42.7%) patients with a high-risk CRS, only 30 underwent color Doppler examination of lower extremity veins, with 15 diagnosed with DVT, resulting in a diagnostic rate of 1.8%. The study found a significant difference in CRS between the non-DVT and DVT groups (4.30 ± 2.71 vs. 9.87 ± 1.46, respectively). Another study also differentiated patients into non-VTE and VTE groups, revealing a significant disparity in CRS between these groups and indicating that VTE can occur even in low-risk patients [64]. A retrospective study [65] was conducted on electrically injured patients at a burn center verified by the ABA and American College of Surgeons over 9 years. This study identified risk factors to calculate CRS at admission and discharge, focusing on outcomes like symptomatic DVT or PE and the timing of these events. Out of 77 patients, the DVT incidence was 6.5%. Those with DVT had significantly higher TBSA burns (27.8% vs. 3.8%), more surgeries (4.8 vs. 0.3), central venous catheter insertion (100.0% vs. 5.3%), longer ventilator use (16.2 d vs. 0.3 d), and longer ICU stays (24.4 d vs. 0.9 d). Notably, their CRS change during hospitalization was markedly higher (18.6 vs. 1.3). Although baseline scores were low, DVT events occurred after multiple risk factors emerged, typically around hospital day 17. Remarkably, DVT incidence rose to 62% in patients whose CRS exceeded 8 points during hospitalization [65].

While this score is useful for assessing VTE risk in burn patients, it doesn’t account for specific factors crucial in burn care, such as inhalation injury and the extent of burn area. Additionally, it includes a maximum of 2 surgeries, whereas severe burn patients often undergo multiple operations. These should be concurrently considered when applying the CRS in burn patients. Considering the current limited evidence specific to burns and data from related fields, we recommend using the CRS for VTE risk assessment in adult burn patients.

### Recommendation 4: It is recommended to assess bleeding risk in hospitalized burn patients receiving anticoagulant/antithrombotic therapy (Strong recommendation, evidence level: low)

Accurate bleeding risk assessment is crucial in VTE management. It’s important to balance reducing thrombosis recurrence risk through anticoagulation against the risk of anticoagulation-induced bleeding. The International Society on Thrombosis and Haemostasis defines bleeding severity and its categories [66]. Bleeding events often occur within the first 3–6 months of anticoagulation, and the risk of major bleeding-induced mortality increases over time [67]. An ideal bleeding risk model should identify reversible bleeding factors, high-risk groups, and estimate individual bleeding risk during anticoagulation therapy.

Currently, there are several VTE bleeding risk prediction models, including the ACCP and European Society of Cardiology models [68–70]. Age, history of (major) bleeding, active malignancy, antiplatelet therapy, anemia, and renal insufficiency were most often used for the prediction of bleeding [71]. For burn patients, complex conditions such as liver and kidney dysfunction, abnormal body weight, systemic inflammation, and malnutrition increase bleeding risk during drug prevention [72–75]. Therefore, continuous bleeding risk assessment, considering specific patient conditions and treatments, is necessary. Clinicians should be aware of the limitations of coagulation tests and adjust management based on the patient’s condition [76]. Accordingly, high bleeding risk does not always contraindicate anticoagulation.

No specialized bleeding risk assessment models for burn patients currently exist, and existing models haven’t been studied in burn populations. Nonetheless, bleeding assessments and monitoring are highly recommended for burn patients, especially those with high-risk factors.

### Recommendation 5: It is recommended to comprehensively diagnose and evaluate DVT in adult burn patients by combining injury condition, clinical manifestations, CRS, high-sensitivity D-dimer, and imaging (Strong recommendation, evidence level: medium)

Burn patients are notably susceptible to DVT. The nature of the injury, such as the cause of the burn, accompanying trauma, or inhalation injury, dictates the patient’s pathophysiological alterations. These include a systemic inflammatory response, stress, endothelial injury, and microthrombosis in soft tissues [77]. Such factors potentially elevate the risk of VTE in burn patients. Particularly in cases of electrical burns, deep burns, and injuries involving inhalation or blast trauma to the lungs, vigilant screening, and evaluation for DVT are crucial [78]. Extensive burns often lead to wound-related complications, including early limb swelling, which, along with bandaging, surgery, and blood exudation, can significantly hinder routine physical examinations and ultrasound procedures, complicating DVT identification [79]. Therefore, a multifaceted approach to diagnosis is recommended.

The CRS, assessing DVT risk based on injury and clinical indicators, is useful for burn patients. For those with a low clinical likelihood of DVT, a high-sensitivity D-dimer test is advised. A negative result largely excludes the possibility of DVT, whereas a positive result necessitates further investigation through ultrasound or venous compression ultrasound (CUS) [79]. In cases where DVT is highly probable or ultrasound is impeded by wounds, alternative imaging methods like computed tomography angiography or magnetic resonance venography (MRV) may be considered [80].

Conventional ultrasound provides direct signs for diagnosing lower extremity DVT. These signs include: (1) presence of solid echoes of varying intensity within the venous cavity, partially or completely filling the vessel; (2) inability or partial inability to compress the lumen with probe pressure; (3) absence of blood flow signals in completely embolized deep veins, as detected by pulse and color Doppler. This includes no increase in blood flow after squeezing the distal limb; (4) in partially embolized veins, pulse Doppler detects a continuous blood flow spectrum at the non-embolized site that does not change with respiration, and color Doppler shows blood flow filling defects. Sometimes, small blood flow is only visible after distal limb compression; and (5) chronic DVT is indicated by vascular wall thickening, lumen narrowing, and the presence of collateral circulation [81].

Several key points in DVT diagnosis among burn patients need emphasis. (1) Due to potential long-term systemic inflammation in burn patients, D-dimer test results alone should not be the sole diagnostic criterion for DVT [82]. (2) Given the complexity of clinical observations and monitoring in burn patients, continuous evaluation with the CRS, D-dimer testing, and imaging is essential [83]. (3) The diagnostic specificity of D-dimer decreases with age; hence, adjusting the cut-off value for elderly patients can enhance specificity [84].

Injury and clinical manifestations indicate that lower limb burns independently increase the risk of DVT [14, 27, 29, 30, 32, 33, 40, 54, 58, 85, 86]. However, burns on other sites (upper limbs, trunk, head, face) do not show a significant correlation with DVT [34]. Regarding burn area, studies suggest that burns covering 40–59% of TBSA carry the highest VTE risk [40]. Inhalation injuries also correlate with increased VTE incidence [87]. Furthermore, during treatment, factors like multiple surgeries, pneumonia, and central venous catheter placement are associated with higher VTE risk [88].

Audu et al. [89] reviewed VTE biomarkers from 2001 to 2019, suggesting high-sensitivity D-dimer as the most relevant for VTE. D-dimer, a degradation product of cross-linked fibrin, rises when thrombi dissolve. A study by Fronas et al. [90] with 913 suspected DVT patients showed a high accuracy of D-dimer testing, with only 1 false negative in 298 cases, indicating a 0.3% failure rate [95% confidence interval (CI) 0.1–1.9%]. The American Society of Hematology (ASH) guidelines for diagnosing VTE [91] recommend high-sensitivity D-dimer testing in patients with low pretest probability of DVT, as it demonstrates superior sensitivity compared with whole-blood or latex semi-quantitative assays. A negative high-sensitivity D-dimer result generally rules out DVT without the need for further imaging, whereas a positive result indicates the need for proximal lower-extremity or whole-leg ultrasonography.

Several studies have demonstrated that in high-risk patients, D-dimer testing alone may miss clinically significant DVT, whereas ultrasonography provides higher diagnostic sensitivity and reliability [92]. For high-risk DVT patients, guidelines suggest starting with ultrasonography, confirming negative results with D-dimer testing, or repeating ultrasonography after a week. In suspected recurrent lower extremity DVT or low probability upper extremity DVT, D-dimer testing is first-line, followed by ultrasonography if positive. For high probability upper extremity DVT, D-dimer testing followed by ultrasonography, or a second ultrasound after a week, is recommended. Conclusively, a negative D-dimer result combined with negative ultrasound, or 2 negative ultrasounds, is needed to rule out upper extremity DVT in high-risk patients [91, 93].

Wahl et al. [94] and Ahuja et al. [33] investigated the utility of D-dimer in screening for DVT among burn patients. Their study involved 30 burn patients, with ultrasounds and D-dimer testing conducted on admission and then weekly until discharge. They identified 11 DVT sites in 7 patients. Notably, unlike trauma patients whose D-dimer levels peak within the first 48 h post-injury, most burn patients did not exhibit elevated D-dimer levels upon admission. However, 86% of those who developed DVT showed elevated levels within the first week, yielding a negative predictive value of 94%. This suggests that a week post-injury, a negative D-dimer result is a strong indicator for excluding DVT in burn patients.

In the second week after burn, systemic complications like wound infection and bacteremia led to hypercoagulable state even in patients without DVT [95, 96]. Although the literature on D-dimer clinical significance in burn patients is limited and its diagnostic value for burn-induced DVT is debated, due to early-stage burn inflammation and coagulation effects, high-sensitivity D-dimer testing is still recommended. A negative high-sensitivity D-dimer test can rule out DVT, and a level above 500 μg/L warrants further investigation.

However, there’s a lack of literature supporting the diagnostic value threshold of high-sensitivity D-dimer in burn patients, particularly considering variables like age and inhalation injury. Moreover, since burn wounds are often located on limbs and are usually bandaged, diagnosing DVT in burn patients should be a comprehensive process. It should involve assessing the injury, clinical manifestations, CRS, high-sensitivity D-dimer results, and imaging evaluations. This holistic approach is strongly recommended for accurate diagnosis and management.

### Recommendation 6: DUS or CUS is recommended as an important method for regular screening and diagnosis of DVT in burn patients (Weak recommendation, level of evidence: low)

Research indicates that the sensitivity of DUS for diagnosing DVT ranges between 88.0% and 98.0%, with a specificity of 97.0–100.0% and an overall accuracy of 97.8%. This high efficacy has made it a standard diagnostic tool for DVT [97–100].

In prospective VTE studies involving burn patients, DVT incidence detected via DUS ranged from 6.08% to 23.3% [40, 101, 102]. This rate is higher compared to diagnoses based solely on symptoms and signs, indicating that clinical symptom-based assessment alone may underestimate DVT in burn patients. This underscores the importance and necessity of using DUS as a primary screening tool for suspected cases in burn patients. Other imaging methods should only be considered when ultrasound diagnosis is unsuitable or inconvenient due to the burn site or poor condition [103].

CUS can identify over 95% of proximal lower extremity venous thrombosis through direct observation of the thrombus, probe compression, extrusion experiments of the distal limb, and Doppler blood flow detection [104]. The key diagnostic indicators for DVT include the inability to compress the vein or the absence of a blood flow signal within the venous lumen [105]. However, its effectiveness is lower for detecting peroneal vein and asymptomatic lower limb muscle DVT [106]. CUS is non-invasive, reproducible, and has largely replaced venography as the preferred DVT diagnostic technique.

Color DUS not only offers anatomical evidence but also provides vascular-related data, with sensitivity and specificity nearing 100%. It’s non-invasive, safe, cost-effective, easily accessible, and can be performed repeatedly, making it the preferred method for detecting DVT in burn patients [90]. However, its accuracy can be moderately affected by burn dressings and tissue edema.

### Recommendation 7: Screening and diagnosis of PE after burns are based on a strategy of suspicion, confirmation, etiology, and risk stratification, combining clinical manifestations, CRS, high-sensitivity D-dimer, and imaging studies (Strong recommendation, evidence level: low)

The clinical manifestations of PE are nonspecific, making it prone to misdiagnosis or underdiagnosis, especially in burn patients who often suffer from inhalation or blast lung injuries [107]. These patients, particularly those with severe burns, frequently experience pulmonary/systemic inflammation and stress due to repeated surgeries, dressing changes, systemic inflammatory responses, and hyper-metabolism, further complicating diagnosis [95]. Acute PE in burn patients can significantly impact oxygenation, wound healing, disease progression, and prognosis [108]. Thus, early screening, evaluation, and management of acute PE in burns are crucial, integrating clinical manifestations, CRS, dynamic high-sensitivity D-dimer testing, and imaging results, considering suspected diagnosis, confirmed diagnosis, etiology, and risk stratification.

Acute PE cannot be excluded solely based on a low clinical likelihood assessment or a negative high-sensitivity D-dimer test [109]. The test’s diagnostic value should be evaluated considering the patient’s age and systemic inflammatory state. In burn patients with a high clinical likelihood assessment, a negative high-sensitivity D-dimer test is not sufficient to rule out PE, and imaging should be performed [110].

Hemodynamic instability in acute PE, presenting as hypotension or shock, must be differentiated from blood pressure drops due to other factors common in burn patients, such as new-onset arrhythmia, hypovolemia, or wound sepsis. Computed tomography pulmonary angiography (CTPA) can delineate the shape and extent of pulmonary artery emboli, differentiate between acute and chronic embolism, measure pulmonary artery and heart chamber diameters, evaluate cardiac function, and assess pulmonary lesions and complications with the pulmonary window [111]. However, CTPA has limitations in evaluating subsegmental pulmonary artery emboli due to its spatial resolution.

Analyzing the cause of acute PE is vital for determining treatment strategy and duration. It’s essential to explore factors contributing to venous stasis, vascular endothelial injury, and hypercoagulable states, including genetic and acquired factors. Identifying reversible risk factors (such as surgery, trauma, medical diseases, and immobilization) in burn patients is crucial. For patients without acute reversible factors, underlying diseases like malignancies, rheumatic immune diseases, endocrine abnormalities, and nephrotic syndrome should be investigated. Young burn patients (< 50 years) with acute PE and no definite acquired risk factors should be screened for thrombophilia and familial VTE [31].

Risk stratification is also recommended for burn patients with confirmed acute PE to guide management. Patients are classified based on hemodynamic status into high-risk (unstable hemodynamics) and low-risk (stable hemodynamics). Hemodynamically stable burn patients with acute PE are further categorized into intermediate-high risk and intermediate-low risk based on the presence of right ventricular dysfunction and/or elevated cardiac biomarkers [112].

### Recommendation 8: CTPA is a preferred method for diagnosing PE after burns (Weak recommendation, evidence level: medium)

CTPA can visually display the shape and location of thrombus in the pulmonary artery and the degree of vascular obstruction. It has high sensitivity and specificity for the diagnosis of PE, is non-invasive and convenient, and has become the first choice for the diagnosis of PE. CTPA can fully visualize the pulmonary artery at the subsegmental level and is the preferred method for pulmonary angiography in patients with suspected PE [101, 113]. The Prospective Investigation on Pulmonary Embolism Diagnosis (PIOPED) II [102] observed a sensitivity of 83% and a specificity of 96% for CTPA in the diagnosis of PE. Negative CTPA had a high negative predictive value for PE in patients with low or moderate clinical likelihood (96% and 89%, respectively), whereas its predictive value decreased to 60% in patients with a high pretest probability. In contrast, the positive predictive value of a positive CTPA was high in patients with intermediate or high clinical likelihood (92–96%) but lower in patients with PE with low pretest probability (58%). Therefore, clinicians should consider further testing in cases where clinical judgment is inconsistent with CTPA findings.

In patients with PE who have low or moderate clinical likelihood, a negative CTPA result is sufficient to exclude PE. However, in patients with a negative CTPA but high clinical likelihood, the need for additional diagnostic evaluation remains uncertain. Although CTPA is the “gold standard” for the diagnosis of PE, its indications should be strictly controlled because it is an invasive examination and carries a possible risk. At the same time, the actual difficulties and feasibility of external examination for burn patients should be fully considered. In addition, the sensitivity and specificity of CTPA in burn patients with acute PE have not been reported. Therefore, CTPA is only recommended as a better diagnostic method, but not a necessary condition. However, other examinations, such as ventilation/perfusion (V/Q) imaging, computed tomography venography, radionuclide examination, and MRV, are not accepted in clinical practice due to varying degrees of complications and the complexity of the examination.

### Recommendation 9: Early closure of the wound, shortening of the course of the disease, and early ambulation training are the most important factors and measures to reduce the occurrence of VTE in burn patients (Strong recommendation, evidence level: medium)

In burn patients, burn wounds, infections, and extended bed rest are factors that increase the risk of developing DVT. A retrospective study of 36,638 burn patients confirmed a significant correlation between burns and the occurrence of VTE [56]. Additionally, a separate retrospective clinical study found that wound infections and extended bed rest (more than 40 d) are independent risk factors for the development of DVT in burn patients [14]. By closing the wound, the likelihood of infection can be reduced, allowing patients to begin early ambulation training [114]. Burns, especially those accompanied by severe conditions such as lower limb burns or inhalation injuries, often require protracted bed rest [115]. Moreover, venous intubation and skin grafting surgery during the treatment process can also extend the patient’s bed rest time, thereby increasing the risk of VTE. However, early ambulation training significantly reduces the incidence of VTE [116], indicating that it is a fundamental preventative measure against VTE [117].

For proximal DVT and high-risk PE, due to the potential risk of thrombus detachment and recurrence, patients should be advised to initiate early ambulation training after sufficient anticoagulant treatment. For remote DVT and low-risk PE, early ambulation training is also recommended.

### Recommendation 10: It is advisable to utilize mechanical prevention methods, and active and passive activities as the primary means of preventing VTE in burn patients daily and those at low risk. Without contraindications, drug prevention should be the preferred option for moderate-high risk VTE patients, which can be complemented by mechanical prevention and/or placement of an inferior vena cava (IVC) filter based on individual circumstances (Strong recommendation, evidence level: medium)

**Fig. 2:**
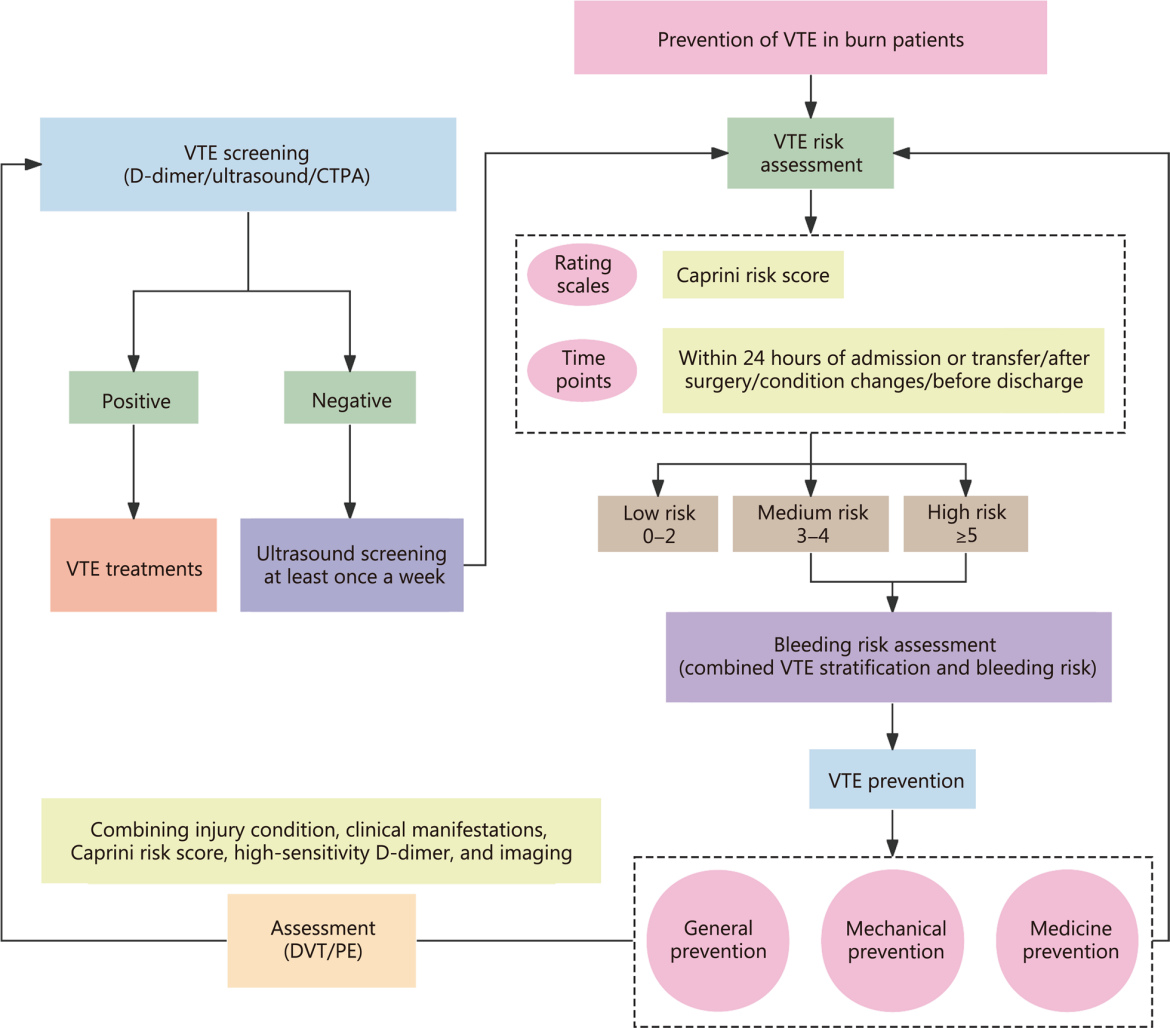
VTE prevention process for burn patients. VTE venous thromboembolism, CTPA computed tomography pulmonary angiography, DVT deep vein thrombosis, PE pulmonary thromboembolism

VTE prevention in burn patients involves a combination of general, mechanical, and medical measures (Fig. 2). General prevention strategies include managing blood lipids and glucose, ensuring adequate fluid intake, elevating affected limbs, minimizing endothelial damage during procedures, early rehabilitation, and functional exercises [12, 78].

A 2021 survey in Australia and New Zealand evaluated VTE prevention practices and outcomes in 3799 adult burn patients. Use of VTE prophylaxis varied between 48.6% and 94.8% of patients. In-hospital mortality occurred in 33 patients (0.87%). After adjusting for confounding factors, patients who received VTE prophylaxis had significantly lower odds of in-hospital death compared with those who did not (adjusted *OR* = 0.21, 95% CI 0.07–0.63; *P* = 0.006) [118].

Mechanical prevention methods, such as graduated compression stockings, intermittent pneumatic compression device (IPCD), and venous foot pump (VFP), enhance venous return in the lower limbs, thus reducing VTE occurrence [119, 120]. Compared to anticoagulants, these methods have a lower bleeding risk, are user-friendly, and are generally well-tolerated by patients.

Low-risk VTE patients should focus on health education and physical activities, with mechanical prevention as an additional option [121]. For moderate- or high-risk patients contraindicated for anticoagulation, mechanical prevention alone is advised [122]. High-risk patients without anticoagulant contraindications should combine mechanical and drug prevention. However, mechanical prevention is not recommended for patients with congestive heart failure, pulmonary edema, lower limb abnormalities (like dermatitis, infection, gangrene, or recent skin grafts), new DVT or thrombophlebitis, severe arteriosclerosis or ischemic vascular diseases, severe limb malformations, or significant lower limb edema [123]. Risk and benefit assessment is crucial in these cases. In high-risk surgical patients, intermittent pneumatic compressions (IPCs) are as effective as anticoagulants in primary outcomes. Using both anticoagulants and compression devices may further reduce VTE risk [124]. Moderate- to high-risk patients benefit significantly from combined drug and mechanical prevention compared to traditional exercise regimens [15]. Thus, mechanical prevention, with its lower bleeding risk and ease of use, is a viable option for low-risk VTE patients. For moderate to high-risk patients, drug prevention remains the primary method, with mechanical prevention as a valuable alternative when pharmacological options are contraindicated.

Routine thromboprophylaxis for burn patients with additional risk factors for VTE is recommended in 2008 ACCP guidelines (Grade 1A) [125]. However, the strength of evidence remains insufficient regarding the comparative effectiveness and safety of pharmacologic versus mechanical strategies for VTE prevention in hospitalized burn patients.

### Recommendation 11: For mechanical prevention, IPCD is recommended as the preferred choice in burn patients without upper or lower limb burn (Strong recommendation, evidence level: low)

IPCDs operate by orderly inflating pressure pumps from the distal to proximal ends, creating a mechanical drainage effect that boosts blood flow and enhances the return of venous and lymphatic fluids. This progressive stress therapy not only alleviates blood stasis and hypercoagulability by stimulating the fibrinolytic system through pressure, but also helps in reducing endothelial cell dysfunction [126, 127].

VTE mechanical prevention equipment includes IPCD, plantar venous pump (foot vein pump, VFP), and gradient pressure elastic socks (graduated compression stockings). In an RCT involving 407 high thrombosis-risk patients (CRS ≥ 11 points) after major surgery, the IPCD group exhibited a notable reduction in asymptomatic DVT [0 vs. 5 cases, relative risk (*RR*) = 0.09, 95% CI 0.01–1.63] [128]. Another trial with 1438 IPCD-treated and 1438 non-IPCD-treated patients revealed that primary outcomes (DVT in the popliteal or femoral vein detected within 30 d or any symptomatic proximal vein DVT confirmed by imaging) occurred in 8.5% of the IPCD group, compared to 12.1% in the non-IPCD group. This equates to an absolute risk reduction of 3.6% (95% CI 1.4–5.8) and a *RR* reduction of 0.69 (95% CI 0.55–0.86) [129]. A single-center randomized controlled study of 108 patients undergoing selective craniotomy for intracerebral lesions found that IPCD use reduced the VTE risk by 75%, significantly lowering potentially fatal VTE incidence in craniotomy patients [130]. In trauma or orthopedic surgery patients, or those with a higher bleeding risk, numerous guidelines strongly advocate IPCD use for mechanical prevention [53, 93, 131, 132]. Compared to anticoagulants alone, the combined use of IPCD can further reduce VTE risk, and bleeding risks associated with IPCD are considerably lower than with anticoagulants [133].

Although direct evidence of IPCD research in adult burn patients is lacking, expert recommendations based on clinical experience, in which IPCD is thought to be convenient and effective, make IPCD a mechanical preventive priority for non-upper or lower limb adult burns (especially calves). Moreover, according to the difference in length, IPCD can be divided into full leg type, lower knee type, and foot bottom type. Limited research in trauma and orthopedic perioperative patients suggests that sub-knee IPCDs are more effective than whole-leg or plantar types in preventing DVT [134]. For adult burn patients, sub-knee types are preferable due to deep vein catheters and the absence of lower limb muscle. In patients with bleeding risks, guidelines recommend mechanical thromboprophylaxis with GCS and/or IPC until the bleeding risk diminishes [53]. Therefore, it is recommended that IPCD be used as a mechanical prevention for VTE in adult burn patients.

### Recommendation 12: We recommend against IVC filter placement as routine prevention for burn patients (Strong recommendation, evidence level: high)

In patients at high risk for proximal DVT, the use of an IVC filter was associated with an increased risk of proximal DVT, yet it did not significantly impact mortality rates [135]. Furthermore, the addition of a retrievable IVC filter to anticoagulation therapy did not demonstrate a reduction in the recurrence of PE when compared to anticoagulation alone [117]. An RCT comparing the effectiveness of sub-vena cava filter implantation combined with anticoagulants vs. anticoagulation therapy alone, found no significant difference in recurrent PE after 6 months of full-dose anticoagulant therapy between the filter and control groups [136].

A study of 558 patients with and without vena cava filters revealed that all post-filter PE cases occurred in patients who had DVT before filter insertion. Moreover, patients without previous VTE events experienced a significantly lower risk of developing IVC thrombosis and PE after filter placement [137]. This indicates that IVC filter use is not warranted in patients without prior VTE who can tolerate anticoagulant therapy, due to the low risk of developing PE.

In the context of burn patients, particularly those with extensive burns, the primary concern is the wound surface. While these patients have a higher risk of VTE, they also face a greater risk of complications like bloodstream infections, which are more prevalent than in standard transwound puncture procedures. The only included cohort study on IVC filter placement in this patient group was at high risk of bias and had significant methodological limitations. It involved only 20 patients and lacked a control group. The high mortality rate observed (9 out of 20 participants) was likely due to multi-organ failure, making it difficult to derive meaningful conclusions about the comparative effectiveness and safety of IVC filters [138].

### Recommendation 13: Low molecular weight heparin (LMWH) is an ideal drug for the prevention and treatment of VTE in burn patients (Strong recommendation, evidence level: high)

Currently, the 3 main intravenous (IV) drugs used for VTE prevention in burn patients are unfractionated heparin (UFH), LMWH, and fondaparinux sodium [139]. LMWH has demonstrated a clear prophylactic effect in cohort studies, showing greater efficacy in preventing VTE compared to UFH [140, 141]. Prospective, RCTs have found no instances of DVT in patients receiving prophylactic LMWH therapy. An RCT involving 100 patients with a 30–60% TBSA burn compared LMWH (enoxaparin 0.5 mg/kg twice daily with a maximum dose of 60 mg/d) throughout the hospital stay in 50 patients, with patients not receiving enoxaparin prophylaxis in the control group. Duplex scanning identified DVT in 4 (8%) out of 50 patients in the control group and none in the LMWH group (*P* = 0.021). Only 1 patient in the enoxaparin group developed mild epistaxis, which resolved spontaneously [33].

The incidence of VTE in patients treated with subcutaneous LMWH injections was 0.9%, lower than the overall incidence of 1.1%. Additionally, the incidence of heparin-induced thrombocytopenia (HIT) was significantly lower with LMWH compared to other drugs [142]. The optimal dosing of enoxaparin for VTE prevention in burn patients is linked to anti-Xa factor activity levels [143]. Previous research indicates that the effective dose of enoxaparin correlates with the patient’s body weight and TBSA [144]. In severe burn patients, inadequate levels of anti-Xa factor may increase VTE risk, so the protocol recommends a prophylactic dose increase from admission, enoxaparin 40 mg twice daily. Monitoring is crucial 4 h after the third dose to ensure anti-Xa factor levels between 0.1 and 0.3 U/ml for effective thrombosis prevention.

Evidence on the use of novel oral anticoagulants in burn patients is limited and lacks a prospective randomized evaluation. While these patients are known to be at risk for venous thrombosis, the *RR* reduction with novel oral anticoagulants is yet to be established. The safety and efficacy of these agents in preventing and treating VTE in burn patients warrant further research. Notably, the 2008 ACCP guidelines [53] recommend using either low-dose UFH or LMWH for VTE prophylaxis as soon as it is safe to do so. Therefore, we recommend LMWH as an ideal drug for the prevention and treatment of VTE in burn patients.

### Recommendation 14: In the absence of contraindications, initial prevention with medication is recommended for burn patients as soon as it is considered safe to do so and continued for as long as the patient remains at risk. The duration depends on clinical risk factors, presentation, and dynamic scoring (Strong recommendation, level evidence: medium)

A meta-analysis encompassing 18 studies and 7515 patients who had symptomatic recurrence of VTE after stopping anticoagulant therapy for at least 3 months revealed significant findings [145]. The risk of recurrent VTE among patients who had completed a minimum of 3 months of anticoagulant therapy for an initial VTE with no apparent cause was 10% in the first-year post-treatment, increasing to 16% at 2 years, 25% at 5 years, and 36% at 10 years. Importantly, 4% of these recurrent VTE events were fatal.

Kearon et al. [146] emphasized that patients with reversible risk factors for VTE, or those with distal DVT without a clear cause, typically require 3 months of treatment. However, for patients with persistent or progressive risk factors like cancer or a second occurrence of proximal DVT or PE without an apparent cause, indefinite treatment is often necessary. The first episode of proximal DVT or PE without a clear cause may be treated for 3 to 6 months or indefinitely. Factors favoring indefinite anticoagulation include being male, PE with proximal DVT, positive D-dimer test after stopping anticoagulation, positive antiphospholipid antibodies, low bleeding risk, and patient preference.

VTE treatment generally requires about 3 months of “active treatment”, followed by continued therapy to prevent new clot formation. The decision between a 3-month treatment course and indefinite therapy depends on various factors. A further meta-analysis of 14 RCTs and 13 cohort studies, including 9982 patients treated with vitamin K antagonists (VKA) and 7220 with direct oral anticoagulants (DOAC), was conducted [70]. This study found a major bleeding incidence of 1.74 events per 100 person-years in the VKA group (95% CI 1.34–2.20) and 1.12 events in the DOAC group (95% CI 0.72–1.62). Over 5 years, the cumulative rate of major bleeding in the VKA group was 6.3% (95% CI 3.6–10.0), highlighting the long-term risk of major bleeding with anticoagulant therapy.

For PE with definitive reversible risk factors, a minimum of 3 months of anticoagulation is advised, with extension based on the patient’s condition (e.g., bedridden status, residual wounds, scars). For persistent PE with risk factors like open or infected wounds, therapy should extend beyond 3 months. Acute DVT with clear reversible risk factors should receive 3 months of anticoagulation, and if risk factors are unclear, therapy should last at least 3 months.

### Recommendation 15: When using different anticoagulant drugs, we recommend to choose different methods for monitoring coagulation function and anticoagulation effect, and to comprehensively evaluate factors such as weight, burn complications, and liver and kidney function to timely adjust the anticoagulant medication and dosage (Strong recommendation, evidence level: medium)

In burn patients, where drug metabolism is altered, enhanced monitoring of coagulation function is crucial. Warfarin typically requires international normalized ratio (INR) monitoring, maintained between 2.0 and 3.0, although some experts recommend targeting INR between 1.6 and 2.5 for individuals aged 75 and older [147]. New oral anticoagulants (NOACs) eliminate the need for routine coagulation monitoring, yet laboratory coagulation assessments remain critical in severe bleeding, thromboembolic events, emergency surgeries, hepatic or renal insufficiency, and suspected drug interactions impacting anticoagulant efficacy and safety [148]. INR is not suitable for monitoring NOACs. For UFH anticoagulation, dynamic monitoring of activated partial thromboplastin time (APTT) is advised, with dosage adjustments based on APTT values to achieve 1.5- to 2.5-times the normal reference value within 24 h [149].

Most patients on LMWH do not require routine clotting function monitoring, but dosage adjustments based on weight are recommended. Adjusting LMWH dosage in conjunction with anti-Xa factor levels is advisable for patients with significant abnormal body weight [body mass index (BMI) < 18.5 kg/m^2^ or BMI ≥ 30 kg/m^2^], and those with creatinine clearance rates between 15 and 30 ml/min. The target anti-Xa factor level should be 0.2–0.4 KU/L.

Higher anti-Xa factor levels are associated with a reduced risk of VTE [150, 151]. For patients on LMWH, using the anti-Xa factor trough level to adjust dosage is advised, especially in high-risk thrombosis cases, and as a result of these findings, an adjusted LMWH dose is recommended by some teams [141, 152–156]. The adjustment of anti-Xa factor drugs closely relates to VTE incidence [157]. However, a retrospective study indicated that a statistically significant reduction in VTE rate and DVT rate but no significant reduction in PE rate after implementation of the anti-Xa titration protocol [158]. In a prospective study of 30 patients with severe burns [TBSA (43 ± 17)%] receiving LMWH, peak anti-Xa activity was decreased on days 5, 10, and 14, this decrease demonstrated an association with elevated nucleosome levels, which was linked to the release of DNA. In this study, 23 (77%) of the 30 patients were affected by heparin resistance, defined as anti-Xa activity < 0.2 U/ml. The authors suggested that monitoring anti-Xa activity with appropriate therapy escalation should be used in patients with severe burns [159].

Following severe burns, coagulation dysfunction, detectable via thromboelastography (TEG), tends to occur and worsen. Dynamic monitoring of TEG can promptly detect procoagulant disorders and hyperfibrinolysis. Among burn patients, those who initially presented with normal coagulation parameters but then developed hypercoagulability during the recovery process, repeat TEG testing would exhibit a stronger tendency towards hypercoagulability [85, 160]. As the severity of burn injuries escalates, so does the need for transfusion. Nevertheless, the employment of TEG or rotational thromboelastometry for hemostatic monitoring can significantly reduce intraoperative transfusion requirements. Furthermore, these 2 monitoring methods effectively predict and guide hemostatic therapy during excision surgery, providing doctors with goal-directed treatment plans [161]. Routine coagulation test is not a reliable indicator of NOAC activity [162]. For necessary drug titration, measuring anti-Xa or anti-IIa factor activity is recommended [148]. A recent cohort study suggests that rapid TEG can establish dose–response curves for multiple NOACs (dabigatran, rivaroxaban, and apixaban), making TEG a valuable tool for studying hemostasis and evaluating reversal strategies in NOAC patients [163].

UFH, excreted extrarenally with a short half-life and easily monitored for anticoagulation, is recommended for patients with severe renal insufficiency to minimize drug accumulation and bleeding risks. In addition, protamine can be given as an antagonist in the event of emergency bleeding after treatment with UFH [164]. Although LMWH tends to accumulate in renal insufficiency, the safety of dalteparin is established in many studies. An observational study comparing dalteparin and UFH in VTE prevention among patients with renal insufficiency [glomerular filtration rate (GFR) < 60 ml/min] showed a significantly lower major bleeding risk in the dalteparin group (1.14% vs. 3.49%, *P* < 0.001) [165]. A subgroup analysis of the PROTECT trial compared VTE prevention in ICU patients with severe renal insufficiency (mean age 61 years, GFR < 30 ml/min) using dalteparin vs. UFH, finding no significant differences in VTE occurrence and bleeding adverse events (*P* = 0.07 and 0.66, respectively) [166]. Therefore, UFH or dalteparin is recommended for patients with severe renal insufficiency. Fondaparinux should be avoided in patients with severe renal insufficiency (GFR < 30 ml/min). Certainly, a minority of patients experiencing HIT exhibit adverse drug reactions, characterized primarily by decreased platelet counts, thrombosis, and skin necrosis, during heparin-induced anticoagulation therapy [167]. HIT is characterized as an antibody-mediated adverse drug reaction triggered by UFH or LMWH. It has been estimated to occur in 0.1–5% of patients receiving therapeutic doses of heparin [167]. Following the onset of HIT, the mortality rate is significantly elevated, necessitating heightened vigilance in clinical practice.

Previous study indicates that underweight patients (BMI < 18.5 kg/m^2^ or weight ≤ 55 kg) require lower VTE prevention drug doses, while obese patients (BMI ≥ 30 kg/m^2^) benefit from higher dosages [168]. Given the heterogeneity in body types and injury severity in burn patients, individualized thromboprophylaxis strategies are necessary [143, 169, 170]. A study evaluating the impact of body weight on anticoagulant dosing found that anti-Xa factor levels correlate with body weight [171]. Obese patients receiving LMWH for acute VTE should base initial LMWH doses on actual body weight, potentially leading to higher anti-Xa factor levels in critically ill patients. A cohort study reported that 12.0% of overweight and 9.1% of underweight patients, respectively, required dosage adjustments to avoid sub-prophylactic anti-Xa factor levels [172, 173]. Weight loss in burn patients often results from acute/chronic comorbidities and age-related metabolic changes, while chronic severe illness can lead to fat accumulation and obesity [12]. Weight considerations are thus essential when administering LMWH for VTE prevention in burn patients.

### Recommendation 16: It is recommended to establish standardized diagnostic and therapeutic procedures for burn patients with VTE risk and stratify for corresponding treatment according to the degree of risk (Strong recommendation, evidence level: medium)

Burn patients with hemodynamic instability (low blood pressure or shock) are considered at high risk for PE [174]. Those with stable hemodynamics are further classified into medium–high and medium-low risk based on right heart function and cardiac biomarkers. Risk stratification for PE can be assisted by clinical examination, electrocardiogram, echocardiogram, cardiac biomarkers, and chest CT, especially for patients with acute massive pulmonary embolism and hemodynamic instability, where rapid risk assessment is crucial [175]. The risk stratification for PE follows 3 steps. First, identify patients with a high risk of early death. The most severe complication of PE is right ventricular overload and dysfunction, which can rapidly lead to circulatory failure and death, necessitating swift diagnosis and timely reperfusion treatment. Second, identify patients with a low risk of complications who can safely receive outpatient treatment, as determined by pulmonary embolism severity index, simplified pulmonary embolism severity index, or Hestia criteria [176–178]. Third, identify patients at intermediate risk. Due to the heterogeneity of this group, it’s important to identify subgroups with a risk of deterioration, who require hospitalization for close monitoring and potential rescue reperfusion and cardiopulmonary resuscitation treatments [112, 179].

### Recommendation 17: During the perioperative period for burn patients, it is advised to: (1) continue prophylactic anticoagulant medications without interruption until operation day except for large excision of eschar or amputations; (2) discontinue antiplatelet therapy according to standard procedures and bridge with LMWH or UFH; and (3) cancel surgery in cases of new, acute VTE (Weak recommendation, evidence level: low)

Patients undergoing anticoagulation therapy for VTE who require invasive interventions should carefully weigh the balance between bleeding and thrombotic risks when considering whether or when to stop anticoagulation, taking into account the time elapsed since the most recent DVT event. For interventions with low bleeding risk, it may be possible to proceed without discontinuing anticoagulation, with the timing of the last dose before the procedure depending on the specific medication. The decision to interrupt anticoagulation during the perioperative period and whether bridging anticoagulation is needed should be made after a thorough assessment of the patient's thromboembolism and bleeding risks. In bridging anticoagulation, the timing, method, and dosage of anticoagulant medications should adhere to guideline recommendations, tailored to the patient’s specific situation, ensuring safety during the perioperative period [180].

In non-burn adult patients, the most unpredictable agents are VKAs, where several days of omission may be required for the INR to drop to normal (non-anticoagulated) levels owing to the long half-life. Omission of LMWHs can be 24 h before the invasive procedure, whereas DOACs should be stopped between 24 and 72 h before the intervention, depending on the specific medication, renal function, and bleeding risk of the procedure [181]. Patients stopping anticoagulation (particularly VKAs) prior to an invasive procedure would require hospital admission for IV infusion of UFH. This has the specific advantage of having a short half-life, allowing maximum anticoagulation right up to the time of the invasive procedure. However, this approach necessitates frequent laboratory monitoring of the APTT. In contrast, DOACs offer a distinct advantage, as they can often be discontinued as briefly as 24 h prior to an invasive procedure. Consequently, bridging anticoagulation is typically obviated [182]. For patients on VKAs, bridging therapy with weight-adjusted doses of LMWH may offer a practical solution to maintaining therapeutic anticoagulation, while avoiding hospital admission [183]. However, in the presence of co-existing specific high-risk indications (such as the presence of some prosthetic heart valves), an IV UFH infusion may still be considered the optimal bridging option.

In non-cardiac surgery with medium–low risk of bleeding, where only a single antiplatelet medication is used, the treatment should not be stopped. Otherwise, aspirin should be discontinued 7–10 d in advance or clopidogrel 5 d before surgery. For dual antiplatelet therapy or high-risk bleeding surgery, regardless of whether it’s cardiac surgery, the medication should be stopped 7–10 d in advance. For patients with coronary bare-metal stents, surgery should be postponed until 6 weeks after stent implantation; if surgery is essential within 6 weeks, continue antiplatelet therapy instead of stopping it 7–10 d before surgery. For patients with coronary drug-eluting stents, surgery should be postponed until 6 months after stent implantation; if surgery is necessary within 6 months, continue antiplatelet therapy rather than stopping it 7–10 d before surgery. Aspirin or low-dose clopidogrel can be restarted 24 h after surgery [184].

**Fig. 3:**
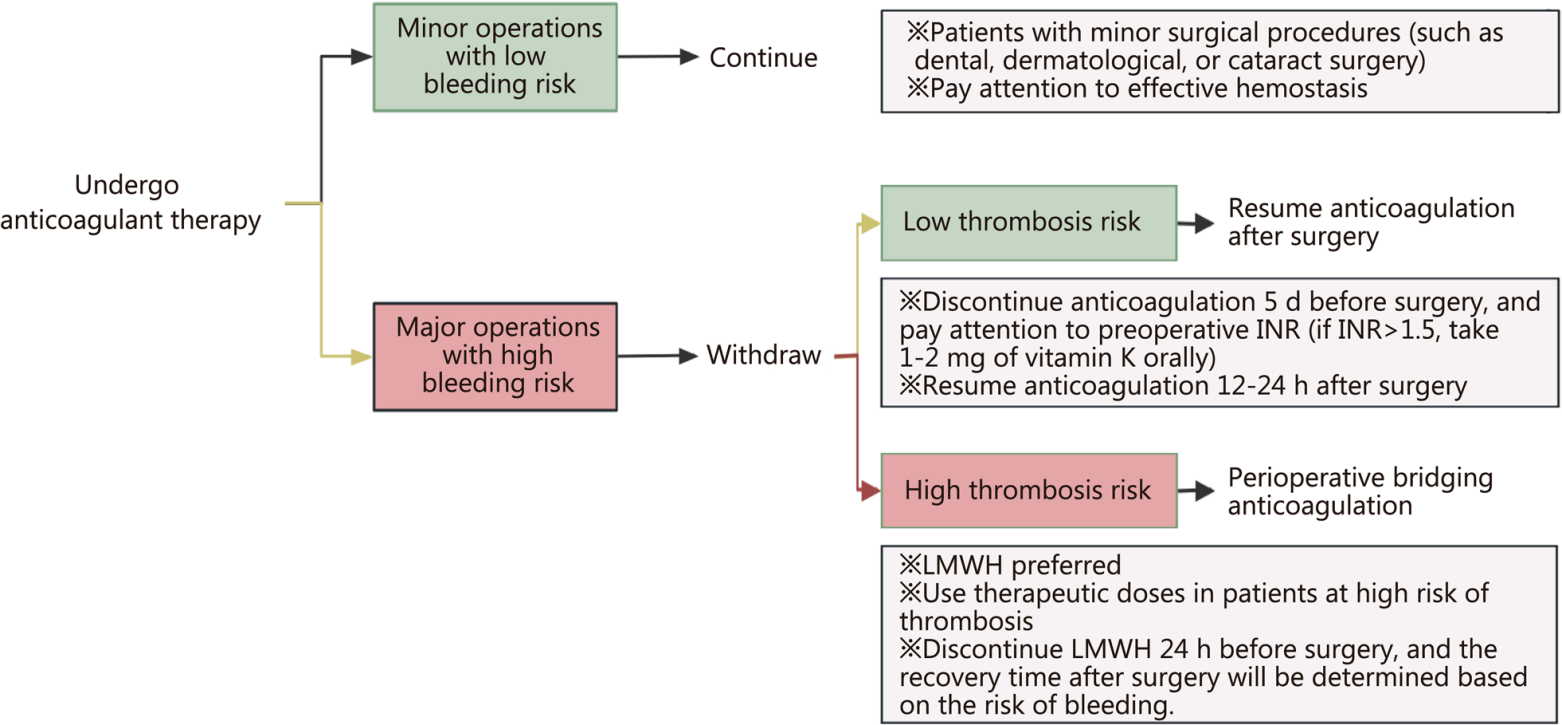
Perioperative anticoagulation strategy for burns. INR international normalized ratio, LMWH low molecular weight heparin

Burn patients (especially those with large area burns) often require multiple surgeries, but burn surgery is considered a superficial operation with a relatively low risk of increasing bleeding except for large excisions of eschar or amputations. Although there is no recommendation for anticoagulation and antiplatelet treatment during the perioperative period in adult burn patients, as a practical clinical problem that burn patients and burn practitioners have to face, experts make the following recommendations based on clinical experience and relevant non-burn inquiry evidence: (1) continue prophylactic anticoagulant medications without interruption until operation day except for large excision of eschar or amputations; (2) antiplatelet therapy should be discontinued according to standard procedures, and bridging with LMWH or UHF should be administered; and (3) surgery should be canceled in the event of new onset VTE [75, 185]. Bridging anticoagulation is an important strategy during the perioperative period, using short-acting drugs in place of long-acting ones minimizes the window for thrombotic events while effectively avoiding unnecessary bleeding, thus reducing the risk of thrombosis and bleeding during the perioperative period [186]. The specific bridging strategies and methods are shown in Fig. 3.

### Recommendation 18: In the case of burn patients who require oral medication, NOACs are prior recommended (Strong recommendation, evidence level: medium)

NOACs encompass direct thrombin inhibitors (DTIs) such as dabigatran [187], and factor Xa inhibitors including rivaroxaban, apixaban, and edoxaban. DTIs bind to thrombin, thereby inhibiting its ability to convert fibrinogen into fibrin, consequently exerting anticoagulant effects [188]. Furthermore, DTIs amplify their anticoagulant action by blocking the thrombin-mediated activation of factors V, VIII, and IX, and impede thrombin-mediated platelet activation, antifibrinolytic activities, and inflammatory processes. Unlike heparin, DTIs are capable of binding with both unbound thrombin and thrombin that is already bound to fibrin. NOACs such as rivaroxaban and apixaban directly act on factor Xa, inhibiting its function in the coagulation process. Characterized by their rapid onset, short half-lives, minimal interactions, and small variations in effective doses, NOACs typically do not require frequent monitoring or dose adjustment [189]. A multitude of RCTs or cohort studies have demonstrated that NOACs are at least as effective and safe as warfarin in VTE prevention, with a lower incidence of bleeding [190–193]. Therefore, NOACs are recommended for elderly patients with chronic severe conditions requiring oral anticoagulation, though no specific medication is preferred over others. The pharmacokinetics of NOACs are influenced by factors such as age, liver, and kidney function. A study had revealed that in patients, the area under the plasma concentration–time curve (AUC) is increased by 41%, likely associated with decreased renal and non-renal drug clearance in older populations [194]. As such, age should be factored into the determination of NOAC dosages. Nonetheless, the exact extent of age’s influence on drug dosage is not clearly established. Renal excretion serves as a major route for the clearance of all NOACs. The level of renal impairment is a significant factor in deciding on the use of NOACs and in selecting the appropriate dosage. For instance, a study indicated that approximately one-third of rivaroxaban is excreted unchanged by the kidneys. For mild renal impairment (creatinine clearance 50–80 ml/min), the AUC is increased to 1.4-fold of that in individuals with normal renal function. For moderate impairment (30–49 ml/min), the AUC increases to 1.5-fold. For severe impairment (15–29 ml/min), the AUC rises to 1.6-fold [195]. Severe renal dysfunction (creatinine clearance < 15 ml/min) is a contraindication for NOACs. The liver is an important organ for the metabolism of many NOACs, with 20% of dabigatran etexilate, 65% of rivaroxaban, 73% of apixaban, and 50% of edoxaban being metabolized hepatically. Impaired liver function also reduces renal clearance of these drugs [196]. A reduction in serum albumin can increase the concentration of unbound drug in the blood. Concurrent coagulopathy, ascites, or a Child–Pugh score of B or C are contraindications for NOAC use [197].

Research on the use of NOACs in burn patients is limited, but numerous high-quality clinical studies in cardiovascular internal medicine or trauma patient care demonstrate that NOACs, such as dabigatran, rivaroxaban, and apixaban, are superior to warfarin in terms of efficacy, with similar or reduced risks of bleeding [198–202]. A retrospective study involving 112 patients treated with NOACs and 373 patients treated with warfarin for anticoagulation in trauma scenarios showed that the overall mortality rate was higher in the warfarin group (10.9%) compared to the NOAC group (6.25%) and the non-anticoagulated control group (5.5%) [203]. Therefore, the use of NOACs is recommended as an oral anticoagulation strategy for burn patients.

### Recommendation 19: It is recommended that in burn patients with life-threatening active bleeding or coagulation dysfunction or heparin resistance, prophylactic pharmacological anticoagulation should be suspended or delayed, and mechanical prevention should be employed (Strong recommendation, evidence level: medium)

Bleeding represents the most severe complication in the prevention and treatment of thrombotic complications [204]. A prospective cohort study enrolled 2745 outpatients treated routinely inanticoagulation clinics, monitoring them from the initiation of oral anticoagulation therapy, with an average follow-up period of 267 d. During this period, there were 153 bleeding complications (7.6 per 100 patient-years). Among these, there were 5 fatal events (all cerebral hemorrhages, 0.25 per 100 patient-years), 23 severe cases (1.1 per 100 patient-years), and 125 minor cases (6.2 per 100 patient-years) [205]. It is advised that anticoagulant therapy in elderly patients should be administered cautiously, and anticoagulation intensity should be closely monitored to minimize the duration of excessive medication. A multicenter retrospective study in Japan found that among 189 patients who experienced massive hemorrhage during anticoagulation therapy, 142 (75%) discontinued anticoagulant use, with temporary and permanent discontinuation accounting for 63 (44%) and 79 (56%) patients, respectively. Within 90 d following the bleeding event, 58 patients (30.7%) died [206].

It is important to note that the risk of severe bleeding persists even after discontinuation of anticoagulants [207]. A meta-analysis [208] encompassing 20 studies (including 17 RCTs) with 8740 patients, over a follow-up period of 13,011 patient-years post-anticoagulation cessation, reported the incidence rates of severe bleeding (*n* = 41) and fatal bleeding (*n* = 7) per 100 patient-years as 0.35 (95% CI 0.20–0.54) and 0.09 (95% CI 0.05–0.15), respectively. The cumulative incidence of severe bleeding 5 years after anticoagulation cessation was 1.0% (95% CI 0.4–2.4). The case fatality rate for severe bleeding after stopping anticoagulant therapy was 19.9% (95% CI 10.6–31.1).

### Recommendation 20: It is recommended to establish a VTE prevention and treatment team for professional burn care and regularly conduct education and training sessions to strengthen the awareness and management of VTE among burn care providers (Strong recommendation, evidence level: low)

Despite the publication of numerous guidelines and consensus statements on VTE, the overall incidence of VTE remains high. This may be attributed to a lack of awareness among healthcare professionals regarding the prevention and treatment of VTE risks in patients [209]. Pingleton et al. [210] formed an interdisciplinary, multidisciplinary team and developed a continuous education program that reduced the incidence rate of VTE by 51% over 18 months. Duff et al. [211] implemented a reform plan that improved adherence to evidence-based guidelines for VTE prevention in hospitalized patients through regular reviews and feedback, documentation assistance, staff education, and various reminders. Within 1 year, the proportion of patients receiving appropriate VTE prevention increased from 49% to 68%. A systematic review by Kahn et al. [212] indicated that interventions such as education, early warning, and multifaceted measures are more effective in preventing VTE than relying solely on healthcare education.

Regarding the specific content of education and training, it is suggested that hospital management departments focus on multidimensional, multilevel, and diverse forms of VTE education and training for burn care professionals [213]. This involves integrating VTE prevention/management protocols and assessment methods into new employee pre-job training; regularly organizing education sessions on VTE cases, key issues, and treatment advances to boost understanding, awareness, and standardized management skills; and promoting academic exchanges to enhance VTE prevention/management. Burn care professionals should prioritize VTE prevention education in daily practice. For patients at risk of VTE and bleeding, healthcare providers should promptly communicate with patients/families, emphasizing VTE risks, prevention knowledge, and patient conditions to facilitate prophylactic measures. Patient education should cover VTE risks/consequences, prevention importance, potential adverse reactions, proper use of prophylactic measures (e.g., limb exercises, anti-thrombotic stockings, IPC pumps), and medication-related knowledge.

In summary, based on our practical experience in relevant disciplines and burn trauma centers, we strongly recommend the establishment of a VTE prevention and treatment team for burn care professionals and the regular conduction of VTE education and training to enhance the concept of VTE prevention and treatment among healthcare providers.

### Recommendation 21: Emphasizing quality control in the prevention and treatment of VTE in burn patients is an important indicator for evaluating the safety and effectiveness of medical care and nursing (Weak recommendation, evidence level: low)

The prevention of VTE has long been considered a key element in healthcare management in many countries. As early as 2010, the National Health Service in the United Kingdom mandated compulsory VTE risk assessment and reporting for all patients in all hospitals [214]. The Agency for Healthcare Research and Quality in the United States regards the incidence of perioperative VTE as a critical patient safety indicator [215]. In China, the former Ministry of Health included “postoperative pulmonary embolism rate” and “postoperative VTE rate” in the “Medical Quality Management and Control Indicators for Tertiary General Hospitals (2011 Edition)” [216]. In 2021, the National Health Commission of China included “improving the standardized prevention rate of VTE” as 1 of the 10 improvement goals in the “National Medical Safety Improvement Objectives” [217].

Quality control in the prevention and treatment of VTE in burn patients is a focus of therapeutic and nursing care quality control in hospitals. It should be considered as one of the important components in the evaluation of hospital quality metrics, departments, and primary care physicians. Regular analysis, evaluation, and assessment of the overall VTE prevention and treatment in burn patients should be conducted, with timely feedback, dynamic improvement, and continuous enhancement. The quality management process includes evaluation of the assessment process, prevention measures, prevention effectiveness, and key quality control indicators.

#### Limitations

It is noteworthy that, while the consensus primarily focuses on VTE in adult burn patients, it does not specifically address special adult populations such as pregnant women, elderly patients, and others. This is primarily because the consensus was primarily aimed at balancing the universality and practicality of the prevention and treatment of VTE in burn patients. Nevertheless, we fully recognize the importance and complexity of VTE in these special populations. In the future, we will conduct more in-depth research and discussions on these special populations, aiming to provide more precise and personalized recommendations for their VTE prevention and treatment. Certainly, it is important to emphasize that most of the recommendations in this consensus are also applicable to these special populations, albeit with the need for careful clinical discrimination.

Moreover, given the scarcity of direct evidence on burn-related VTE, we actively drew upon the rich experience and evidence from other disciplines, particularly the surgical system, in VTE prevention and treatment during the consensus development process. This is primarily based on the similarity in pathogenesis and the close interdisciplinary connections. Through extensive discussions and comprehensive analysis among experts, we believe that this evidence can provide valuable references and insights for the prevention and treatment of VTE in burn patients.

## Conclusions

This consensus includes a total of 21 recommendations on the prevention, diagnosis and management of VTE in adult burn patients, which is the first comprehensive coverage of the clinical aspects of VTE in adult burns, and fills a gap in this field at home and abroad, and can improve the prognosis and quality of life of these patients ultimately.

## Supplementary Information

**Additional file 1**. Consultant experts. **Table S1** Basic information of the participating experts. **Table S2** Concentration, coordination, and authority of the consensus content.

## Data Availability

Not applicable.

## Abbreviations

ABA: American Burn Association
ACCP: American College of Chest Physicians
APTT: Activated partial thromboplastin time
AUC: Area under curve
BMI: Body mass index
CI: Confidence interval
CRS: Caprini risk score
CTPA: Computed tomography pulmonary angiography
CUS: Compression ultrasound
CV: Coefficient of variation
DOAC: Direct oral anticoagulants
DTIs: Direct thrombin inhibitors
DUS: Doppler ultrasound
DVT: Deep vein thrombosis
GFR: Glomerular filtration rate
GRADE: Grading of Recommendations Assessment, Development and Evaluation
HIT: Heparin-induced thrombocytopenia
ICU: Intensive care unit
INR: International normalized ratio
IPCD: Intermittent pneumatic compression device
IPCs: Intermittent pneumatic compressions
IV: Intravenous
IVC: Inferior vena cava
LMWH: Low molecular weight heparin
MRV: Magnetic resonance venography
NOACs: New oral anticoagulants
OR: Odds ratio
PE: Pulmonary thromboembolism
RCT: Randomized controlled trial
RR: Relative risk
TBSA: Total body surface area
TEG: Thromboelastography
UFH: Unfractionated heparin
VFP: Venous foot pump
VKA: Vitamin K antagonists
VTE: Venous thromboembolism
