# Supplementary material for: Chinese expert consensus on prevention, diagnosis, and management of venous thromboembolism in adult burn patients (2024)
Source: Mil Med Res. 2025 Nov 2;12:74. doi: 10.1186/s40779-025-00653-9 (PMC12581310; doi:10.1186/s40779-025-00653-9)
Supplement: Supplementary file 1 — Additional file 1. Consultant experts. Table S1 Basic information of the participating experts. Table S2 Concentration, coordination, and authority of the consensus content. [file 40779_2025_653_MOESM1_ESM.pdf]

Consortia

Burn and Trauma Branch of the Chinese Geriatric Medical Association, Critical Care Group of Burn Surgery Branch of the Chinese Medical Association

Consultant experts (in no particular order)

Xiao-Bing Fu (Key Laboratory of Wound Repair and Regeneration of PLA, the Fourth Medical Center of PLA General Hospital, Beijing 100048, China), **Yong-Hua Sun** (Department of Burn, Beijing Jishuitan Hospital, Capital Medical University, Beijing 100035, China), **Yue-Sheng Huang** (Department of Wound Repair, Institute of Wound Repair and Regeneration Medicine, Southern University of Science and Technology Hospital, Southern University of Science and Technology School of Medicine, Shenzhen 518055, Guangdong, China), **Guo-Zhong Lv** [Department of Wound Repair, Nanjing University of Chinese Medicine Wuxi Integrated Traditional Chinese and Western Medicine Hospital (Affiliated Hospital of Jiangnan University), Wuxi 214122, Jiangsu, China], **Guang-Hua Guo** (Medical Center of Burn Plastic and Wound Repair, the First Affiliated Hospital of Nanchang University, Nanchang 330006, China), **Xu-Lin Chen** (Department of Burns and Wound Repair, the First Affiliated Hospital of Anhui Medical University, Hefei 230022, China), **Shu-Liang Lu** (Department of wound healing, RuiJin Hospital, School of Medicine, Shanghai Jiaotong University, Shanghai 200025, China), **Jing-Ning Huan** (Department of Burn and Plastic Surgery, Ruijin Hospital, School of Medicine, Shanghai Jiaotong University, Shanghai 200019, China), **Pi-Hong Zhang** (Department of Burns and Plastic Surgery, Xiangya Hospital, Central South University, Changsha 410008, China), **Ke Tao** (Department of Wound Repair, Wound Repair and Regenerative medicine Center, the First Affiliated Hospital of Wenzhou Medical University, Wenzhou 325000, Zhejiang, China), **Zong-Yu Li** (Department of Burns, Harbin Fifth Hospital, Harbin 150040, China), **Hong-Yan Zhang** (Medical Center of Burn Plastic and Wound Repair, the First Affiliated Hospital of Nanchang University, Nanchang 330006, China), **Hong-Wei Liu** (Department of

Burns and Plastic Surgery, the First Hospital of Jinan University, Guangzhou 510630, China), **Yu-Feng Jiang** (Department of Tissue Regeneration and Wound Repair, PLA General Hospital, Beijing 100853, China), **Ding-Hong Min** (Medical Center of Burn Plastic and Wound Repair, the First Affiliated Hospital of Nanchang University, Nanchang 330006, China), **Cheng-De Xia** (Department of Burns, Zhengzhou First People's Hospital, Zhengzhou 450004, China), **Qing-Fu Zhang** (Burns and Wound Repair Center, the Third Hospital of Hebei Medical University, Shijiazhuang 050035, China), **Shi-Hui Zhu** (Department of Burns and Plastic Surgery, Shanghai Children's Medical Center, Shanghai Jiaotong University, Shanghai 200127, China), **Yi Liu** (Burn, Plastic & Wound Repair Surgery, Lanzhou University Second Hospital, School of Medicine, Lanzhou University, Lanzhou 730030, China), **Jia-Ping Zhang** (Department of Plastic Surgery, State Key Laboratory of Trauma and Chemical Poisoning, the First Affiliated Hospital, Army Medical University, Chongqing 400038, China), **Yu-Ming Shen** (Department of Burns, Beijing Jishuitan Hospital, Capital Medical University, Beijing 100035, China), **Biao Cheng** (Department of Burn & Plastic Surgery, General Hospital of Southern Theatre Command of PLA, Guangzhou 510010, China), **Wei-Guo Xie** (Institute of Burns, Tongren Hospital of Wuhan University&Wuhan Third Hospital, Wuhan 430060, China), **Guang-Ping Liang** (Chinese Journal of Burns and Wounds, Chongqing 400038, China), **Feng Zhu** (Department of Critical Care Medicine, Shanghai East Hospital, School of Medicine, Tongji University, Shanghai 200120, China), **Shao-Wen Cheng** (Department of Wound Repair, the First Affiliated Hospital of Hainan Medical University, Haikou 570102, China), **Hua Zhou** (Burn Plastic Wound Repair Surgery, Ganzhou Hospital of Guangdong Provincial People's Hospital, Ganzhou Municipal Hospital, Ganzhou 341000, China), **Ye-Xiang Sun** (Department of Burns and Wound Repair, the First Affiliated Hospital of Anhui Medical University, Hefei 230022, China), **Da-Hai Hu** (Department of Burns and Cutaneous Surgery, Xijing Hospital, Air Force Medical University, Xi'an 710032, China), **Chi-Yu Jia** (Department of Burns and Plastic and Cosmetic Surgery, Burns Plastic Surgery and

Wound Repair Center, the First Hospital Affiliated to Hengyang Medical College, Nanhua University, Hengyang 421001, Hunan, China), **Wei Ding** (the First Affiliated Hospital of Wannan Medical College, Wuhu 241000, Anhui, China), **Yi Zhang** (Department of Burn and Plastic Surgery, Department of Wound Repair Surgery, Affiliated Hospital of Nantong University, Nantong 226001, Jiangsu, China), **Xiao-Jian Li** (Department of Burns and Plastic Surgery, Guangzhou Red Cross Hospital, Guangzhou 510220, China), **Du-Yin Jiang** (Department of Plastic and Burns Surgery, the Second Hospital of Shandong University, Jinan 250033, China), **Qin Zhou** (Department of Burns and Cutaneous Surgery, Xijing Hospital, Air Force Medical University, Xi'an 710032, China), **Liang Zheng** (School of Medicine, Tongji University, Shanghai 200120, China), **Hai-Tao Ren** (Department of Vascular Surgery, the Second Affiliated Hospital, Zhejiang University School of Medicine, Hangzhou 310009 China).

Table S1 Basic information of the participating experts

**Parameters**	**Number (%)**
Recovery rate (%)	100 (100.0)
Response rate (%)	100 (100.0)
Age	
< 45 years	6 (16.2)
45 – 60 years	20 (54.1)
> 60 years	11 (29.7)
Sex	
Male	34 (91.9)
Female	3 (8.1)
Education	
Doctor of medicine	32 (86.5)
Master	5 (13.5)
Professional title	
Senior professor	33 (89.2)
Vice-senior professor	4 (10.8)
Years of professional experience	
< 20 years	10 (27.0)
20 – 30 years	12 (32.4)
> 30 years	15 (40.6)
Professional fields	
Burns/Wound repair/Plastic surgery	35 (94.6)
Intensive Care Unit	1 (2.7)
Medical Journal Editorial Board	1 (2.7)

Table S2 Concentration, coordination, and authority of the consensus content

Consensus	Mean ± SD	Full score ratio (%)	Coefficient of variation	Coordination coefficient	Judgment coefficient	Familiarity coefficient	Authority coefficient
VTE is a prevalent complication following burn injuries, particularly in patients with extensive, critical burns	8.90 ± 0.35	91	0.04	0.28	0.83 ± 0.05	0.85 ± 0.04	0.84 ± 0.05
Age, obesity, extensive burns, deep venous catheters, prolonged ICU stay, immobility, wound infection, operations, inhalation injuries/trauma are primary risk factors	8.70 ± 0.52	78	0.06	0.29	0.82 ± 0.06	0.84 ± 0.05	0.83 ± 0.05
CRS is recommended for VTE risk assessment in hospitalized burn patients	8.65 ± 0.57	75	0.07	0.27	0.81 ± 0.06	0.83 ± 0.05	0.82 ± 0.05
Assess bleeding risk in hospitalized burn patients receiving anticoagulant/antithrombotic therapy	8.55 ± 0.76	68	0.09	0.28	0.80 ± 0.07	0.82 ± 0.06	0.81 ± 0.06
Comprehensive diagnosis of DVT using injury condition, clinical manifestations, CRS, high-sensitivity D-dimer, and imaging	8.65 ± 0.57	75	0.07	0.30	0.82 ± 0.06	0.84 ± 0.05	0.83 ± 0.05
Ultrasound or CUS for regular screening and diagnosis of DVT in burn patients	8.40 ± 0.76	68	0.09	0.28	0.81 ± 0.07	0.83 ± 0.06	0.82 ± 0.06
PE screening strategy: suspicion, confirmation, etiology, risk stratification, combining clinical, CRS, D-dimer, and imaging	8.60 ± 0.76	68	0.09	0.27	0.81 ± 0.07	0.82 ± 0.06	0.81 ± 0.06
CTPA is preferred for diagnosing PE after burns	8.42 ± 0.57	75	0.07	0.29	0.82 ± 0.06	0.83 ± 0.05	0.82 ± 0.05
Early wound closure, disease course shortening, and early ambulation reduce VTE	8.65 ± 0.57	75	0.07	0.30	0.82 ± 0.06	0.84 ± 0.05	0.83 ± 0.05
Mechanical prevention, active/passive activities, drug prevention for moderate-high risk, and optional IVC filter	8.65 ± 0.57	75	0.07	0.29	0.82 ± 0.06	0.84 ± 0.05	0.83 ± 0.05
IPCD recommended for mechanical prevention in patients without limb burn	8.55 ± 0.78	68	0.09	0.28	0.81 ± 0.07	0.83 ± 0.06	0.82 ± 0.06
Against routine vena cava filter placement	8.95 ± 0.30	94	0.03	0.32	0.85 ± 0.04	0.87 ± 0.03	0.86 ± 0.04
LMWH is ideal for the prevention and treatment of VTE	8.95 ±	94	0.03	0.32	0.85 ± 0.04	0.87 ± 0.03	0.86 ± 0.04

Consensus	Mean ± SD	Full score ratio (%)	Coefficient of variation	Coordination coefficient	Judgment coefficient	Familiarity coefficient	Authority coefficient
	0.30						
Initial medication prevention as soon as safe, duration per risk factors, and scoring	8.65 ± 0.57	75	0.07	0.29	0.82 ± 0.06	0.84 ± 0.05	0.83 ± 0.05
Adjust anticoagulation based on drug type, body weight, complications, liver/kidney function	8.65 ± 0.57	75	0.07	0.29	0.82 ± 0.06	0.84 ± 0.05	0.83 ± 0.05
Establish standardized diagnostic and therapeutic procedures for VTE risk stratification	8.65 ± 0.57	75	0.07	0.29	0.82 ± 0.06	0.84 ± 0.05	0.83 ± 0.05
Perioperative: continue prophylaxis, bridge antiplatelet, cancel surgery for new VTE	8.40 ± 0.76	68	0.09	0.27	0.81 ± 0.07	0.82 ± 0.06	0.81 ± 0.06
Oral NOACs are recommended when required	8.65 ± 0.57	75	0.07	0.29	0.82 ± 0.06	0.84 ± 0.05	0.83 ± 0.05
Suspend/delay anticoagulation in bleeding/coagulation dysfunction/heparin resistance, use mechanical prevention	8.65 ± 0.58	75	0.07	0.29	0.82 ± 0.06	0.84 ± 0.05	0.83 ± 0.05
Establish a VTE prevention/treatment team, regular education for burn care providers	8.50 ± 0.76	68	0.09	0.28	0.81 ± 0.07	0.83 ± 0.06	0.82 ± 0.06
Emphasize quality control in VTE prevention/treatment as an evaluation indicator	8.50 ± 0.78	68	0.09	0.28	0.81 ± 0.07	0.83 ± 0.06	0.82 ± 0.06

VTE venous thromboembolism, *ICU* intensive care unit, *DVT* deep vein thrombosis, *CRS* Caprini risk score, *CUS* compression ultrasound, *CTPA* computed tomography pulmonary angiography, *PE* pulmonary thromboembolism, *IVC* inferior vena cava, *IPCD* intermittent pneumatic compression device, *LMWH* low molecular weight heparin, *NOACs* new oral anticoagulants, *SD* standard deviation
